# Editorial: Innovators in chemistry: 2022

**DOI:** 10.3389/fchem.2023.1352328

**Published:** 2024-01-05

**Authors:** Aleksandar Kondinski

**Affiliations:** ^1^ Cambridge Centre for Advanced Research and Education in Singapore Ltd, Singapore, Singapore; ^2^ University of Cambridge, Cambridge, United Kingdom

**Keywords:** chemical innovation, chemical technology, synthesis, materials, life science

**FIGURE 1:**
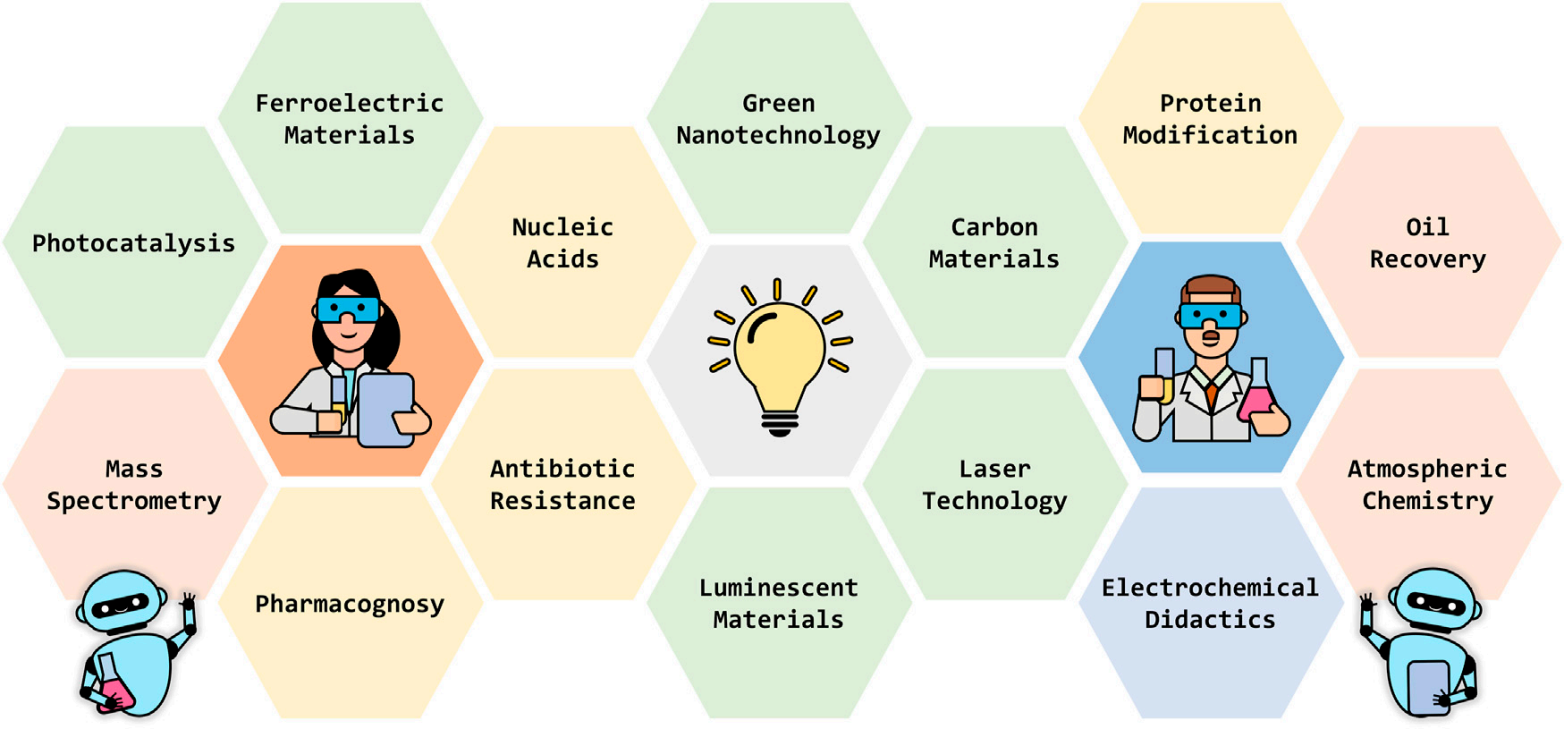
Overview of chemical domains covered in the innovators in chemistry 2022.

The “Innovators in Chemistry 2022” edition showcases the dynamic evolution and critical importance of chemistry in addressing complex challenges across various fields, emphasizing its role in driving sustainable advancements. This issue, curated by a number of young guest editors, assembles a diverse group of both established and emerging researchers whose work significantly impacts the field. Featuring 14 meticulously crafted articles by 80 researchers, the Research Topic spans a wide array of topics in chemical science, including nucleic acid structures, antibiotic resistance, pharmacognosy, green nanotechnology, photocatalysis, ferroelectric materials, protein modification, carbon materials, mass spectrometry, laser technology, luminescent materials, atmospheric chemistry, oil recovery technology, and didactical electrochemistry (Figure 1). It offers a comprehensive view of current trends and future innovations through original research, reviews and opinion articles. This edition not only celebrates recent advancements but also seeks to inspire ongoing exploration and progress in chemistry. Conclusively, “Innovators in Chemistry 2022” highlights the collaborative and intellectual contributions of its participants in steering the future of this central science. The guest editorial team expresses deep gratitude to each author, reviewer, and reader for their invaluable involvement in advancing innovative solutions and understanding of chemistry.

In this edition, we explore a number of exceptional research directions in life sciences. Monsen presents a comprehensive review on G-quadruplexes (G4s), that is four-stranded nucleic acid structures forming in guanine-rich regions of chromatin, particularly in gene promoters. His work discusses the significance of G4s as epigenetic elements in gene transcription and their potential as indirect drug targets in cancer therapies, advocating for research focused on higher-order G4s for more selective molecular targeting, using the human telomerase reverse transcriptase (hTERT) core promoter G-quadruplex as a case study (Monsen). Complementing this, Krco et al. delve into the challenge of antibiotic resistance, with a special focus on metallo-*β*-lactamases (MBLs) that inactivate *β*-lactam antibiotics. Their review emphasizes the structure-function relationships of B3-type MBLs, noting their active site diversity and the potential for broader-range inhibitors, as suggested by the inhibition of one B3-type MBL by clavulanic acid (Krco et al.). In a distinct but equally innovative domain, Myklebust et al. report the development of potent bisubstrate inhibitors targeting NAA80, an enzyme crucial for actin N-terminal acetylation in eukaryotic cells. Their optimized compound, CoA-Ac-EDDI-NH_2_, combines coenzyme A and a tetrapeptide amide, showing high efficacy and promising insights into the roles of actin and the regulatory functions of NAA80 (Myklebust et al.). Lastly, Shaheen et al. provide a comprehensive analysis of *Xanthium strumarium L.* (XSL) foliage, exploring its antioxidant and anti-diabetic properties. They demonstrate that the ethyl acetate fraction of XSL, with its high phenolic concentration, significantly reduces blood glucose levels in diabetic mice, indicating a link to its traditional use in diabetes treatments (Shaheen et al.).

The landscape of advanced materials technology is rapidly evolving, as demonstrated by a series of recent studies. Zidane et al. exemplify this evolution through their work on eco-friendly nanoparticle synthesis using Purslane leaf extract, marking a significant departure from traditional hazardous chemical methods towards environmentally sustainable practices. This theme of sustainability is further explored by Lu et al., who unveil the potential of transition metal single-atom catalysts supported by red phosphorus to enhance photocatalytic hydrogen production, a crucial aspect in the search for clean energy solutions. The innovative use of materials continues in the realm of biological imaging, as Thornton et al. develop unique macrocyclic lanthanide complexes, opening new avenues in advanced imaging techniques. Expanding the boundaries of material science, Mills et al. delve into the complex effects of doping on the ferroelectric and dielectric properties of barium titanate, emphasizing the complex relationship between material composition and its properties. Concurrently, Ni et al. pose their viewpoint on the advancements in carbon-based materials, highlighting the diverse applications of porous carbon in fields ranging from adsorption and catalysis to energy storage, crucial for tackling current energy and environmental challenges. Finally, the innovative synthesis of two-dimensional transition metal dichalcogenides using laser-assisted techniques, as detailed by Wang et al., showcases a method that promises to trailblaze flexible electronics with its precision and dynamic properties. Collectively, these studies not only reflect the current state of advanced materials technology but also chart a path for future research and applications in this rapidly evolving field.

Recent advancements in chemical analysis and control technologies are highlighted in a series of studies, each focusing on different applications and implications. Reynaud et al. present a significant leap in the field of mass spectrometry (MS) with their development of a compact nano-electro-mechanical system (NEMS) based MS, designed for ultra-high mass analytes. This prototype, operating under higher pressures without compromising on particle focusing or mass measurement quality, demonstrates an improved capability in nanoparticle analysis, a crucial advancement for MS technology. Complementing this, Dash et al. delve into atmospheric chemistry, investigating the formation of methanimine in the troposphere. By exploring the reaction dynamics of the aminomethyl radical with oxygen and considering the influences of ammonia and water, their study sheds light on the complex chemical processes occurring in our atmosphere, contributing to a deeper understanding of tropospheric compound formation. Du et al. address a practical challenge in the oil recovery industry, focusing on the flow resistance characteristics of steam injection in horizontal oil wells. Their experimental approach to evaluating pressure loss and optimizing steam distribution offers a refined method for improving oil recovery, showcasing the application of fluid dynamics and pressure control in an industrial setting.

In response to the increasing importance of electroanalytical chemistry increasing significance, Espinoza-Montero et al. developed a didactical guide on generating hydroxyl radicals ∙OH using boron-doped diamond (BDD) in electrochemical oxidation processes. This educational resource elucidates key aspects such as BDD activation, electrolyte response analysis, and overpotential determination for ∙OH production through Tafel plots and voltammetry, along with strategies for radical entrapment. Additionally, their study extends to the practical application of these methods for the efficient and cost-effective degradation of amoxicillin, thereby showcasing the distinct electronic characteristics and stability of BDD in redox reactions (Espinoza-Montero et al.).

On behalf of the involved guest editors and myself, I express our gratitude to the contributors and reviewers who played a vital role in shaping this Research Topic. Gratitude is also extended to the Frontiers team for their invaluable support, making this endeavor both enjoyable and timely. We hope this edition delights the readers of Frontiers in Chemistry and showcases the significant advancements. It is our aspiration that this Research Topic will spark interest, initiate new thoughts, and shed light on uncharted areas of chemical innovation.

